# Effects of Scale on Multimodal Deixis: Evidence From Quiahije Chatino

**DOI:** 10.3389/fpsyg.2020.584231

**Published:** 2021-01-12

**Authors:** Kate Mesh, Emiliana Cruz, Joost van de Weijer, Niclas Burenhult, Marianne Gullberg

**Affiliations:** ^1^Lund University Humanities Lab, Lund University, Lund, Sweden; ^2^Department of Anthropology, Centro de Investigaciones y Estudios Superiores en Antropología Social (CIESAS-CDMX), Mexico City, Mexico; ^3^Centre for Languages and Literature, Lund University, Lund, Sweden

**Keywords:** deixis, pointing, multimodality, indicating, demonstratives, Mesoamerica

## Abstract

As humans interact in the world, they often orient one another's attention to objects through the use of spoken demonstrative expressions and head and/or hand movements to point to the objects. Although indicating behaviors have frequently been studied in lab settings, we know surprisingly little about how demonstratives and pointing are used to coordinate attention in large-scale space and in natural contexts. This study investigates how speakers of Quiahije Chatino, an indigenous language of Mexico, use demonstratives and pointing to give directions to named places in large-scale space across multiple scales (local activity, district, state). The results show that the use and coordination of demonstratives and pointing change as the scale of search space for the target grows. At larger scales, demonstratives and pointing are more likely to occur together, and the two signals appear to manage different aspects of the search for the target: demonstratives orient attention primarily to the gesturing body, while pointing provides cues for narrowing the search space. These findings underscore the distinct contributions of speech and gesture to the linguistic composite, while illustrating the dynamic nature of their interplay.

Abstracts in Spanish and Quiahije Chatino are provided as appendices.

Se incluyen como apéndices resúmenes en español y en el chatino de San Juan Quiahije. Son^G^ ktyi^C^ re^C^ in^H^, ngyaq^C^ ska^E^ ktyi^C^ no^E^ nda^H^ son^B^ na^F^ nga^J^ no^I^ ngyaq^C^ lo^E^ ktyi^C^ re^C^, ngyaq^C^ ran^F^ chaq^E^ xlya^K^ qo^E^ chaq^F^ jnya^J^ no^A^ ndywiq^A^ renq^A^ Kchin^A^ Kyqya^C^.

## 1. Introduction

Language users regularly *indicate* entities—that is, they reorient attention to particular spaces, and prompt a search for entities within those spaces. The act of indicating is performed with apparent ease, and yet it is strikingly intricate, often involving the combination of speech and gesture to manage attention. The complexity of indicating, and especially its multimodal character, have drawn interest in the cognitive sciences, with special consideration given to the combination of demonstrative expressions and deictic gestures. Yet studies of these two strategies have mainly explored their use in laboratory settings, asking how pointing and demonstratives are combined to indicate manipulable objects, often within or just outside of the speaker's and addressee's reach. As a result, we know surprisingly little about how demonstratives and deictic gestures are coordinated to manage attention in large-scale space and in actual usage. Here, we present a first study of multimodal indicating that takes into account the effect of scale, and focuses on multimodal indicating in large-scale space in particular. We study this phenomenon in a naturalistic setting, considering how speakers indicate named places and participate in familiar direction-giving practices. Our study is performed with speakers of Quiahije Chatino, an indigenous language of Mexico in which multiple features of demonstrative use and pointing practice have already been documented, facilitating a closer study of their combination in multimodal indicating acts. We begin the paper by reviewing the theoretical and empirical background to research on indicating, and then contextualize the placement of our project in the Quiahije Chatino-speaking community, before turning to the current empirical study.

## 2. Background

### 2.1. Elements of an Indicating Event

To *indicate* is to direct attention to something by creating a connection to it in space and/or time (Peirce, 1955; Clark, 1996, 2003, 2020). A typical act of indicating involves a *sender* (a speaker or signer, depending on the language modality), an *addressee* whose attention can be managed (cf. Burenhult, 2018), an object for their attention, variously called a *referent* or *target* (cf. Clark and Bangerter, 2004; Talmy, 2018), and crucially a spoken or embodied *sign* that evokes a delimited search domain in which the target can be found. Some indicating acts draw a connection to an imaginary target (Bühler, 1934; Levy and McNeill, 1992; Cooperrider, 2014; Stukenbrock, 2014; Rocca and Wallentin, 2020) or to a target present in speech rather than in the spatiotemporal context (Levy and McNeill, 1992). A common act of indicating—often deemed prototypical—draws attention to a concrete entity in the real-world space surrounding the sender and addressee: this kind of *exophoric* indicating will be our focus here (Fillmore, 1982; Diessel, 1999; Levinson, 2004; Fricke, 2014).

Spoken languages have a specialized set of signs for indicating—demonstrative expressions, such as English's *this* and *that, here* and *there*. In gesture and in sign languages, the same function is served by deictic movements including pointing (Kendon and Versante, 2003; Kita, 2003; Cooperrider and Mesh, in press). Both of these indicating behaviors manage an addressee's attention and delimit the search domain for the target along some dimension(s), such as direction or distance. Both behaviors also invoke other features that may further delimit the search domain, or characterize the participants' perceptual and attentional relationship to the target (Burenhult, 2003; Jungbluth, 2003; Küntay and Özyürek, 2006).

No matter the modality in which it is performed, indicating demands that an addressee attend to the intended target in the search domain. This task is facilitated if the addressee has a conception of the scale of the domain: an expression like *here* might evoke the space on a microscope slide or the expanse of a galaxy, and attention may be aimed quite differently in search domains at different scales.

Some investigations of spatial indicating have explicitly invoked the notion of scale, asking whether speakers have specialized strategies for indicating targets within their reach (cf. Kemmerer, 1999; Wilkins, 1999a; Coventry et al., 2008, 2014; Gudde et al., 2016), within delimited spaces where ongoing activities are taking place (Wilkins, 1999b, 2018), and at “expanded” scales, including “landscape scale” or “large-scale geographical space” (cf. Wilkins, 1999b, 2018; Bril, 2004; Ozanne-Rivierre, 2004; Burenhult, 2008; Schapper and San Roque, 2011). These studies are categorized into two types: research in highly controlled laboratory experimental settings, where the scales in question are typically encompassed within the space of a room, and elicitation studies that consider strategies across a greater range of scales, but report speaker intuitions rather than observed indicating behaviors. As a consequence, we know little about how people indicate targets at different scales in natural communication contexts.

### 2.2. Demonstratives and Scale

Demonstratives are a closed grammatical class of expressions specialized for indicating: they manage the addressee's attention by inviting a search for some target, and evoking a search domain in which the target can be found. They are deictic, relating the search domain to either of the speech act participants (speaker and addressee) or to the broader speech situation (see, e.g., Burenhult 2008, p. 100). To delimit the search space, demonstratives have traditionally been said to encode paradigmatic oppositions (Himmelman, 1996) of distance (Anderson and Keenan, 1985; Diessel, 1999, 2014; Dixon, 2003). An increasing number of studies finds that demonstrative oppositions are better characterized in terms of participants' shared knowledge and context, rather than in terms of distance (e.g, Laury, 1997; Enfield, 2003, 2018; Piwek et al., 2008; Jarbou, 2010; Peeters et al., 2015b; Peeters and Özyürek, 2016; Rocca et al., 2019), though distance has a role to play in shaping that context (cf. Burenhult, 2003, p. 365; 2018, p. 367).

Talmy (1988, p. 168–169) observes that demonstrative oppositions–whatever their semantic encodings–can operate at multiple scales. He provides an example in the sentences in (1):


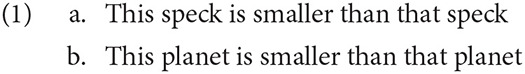


This observation about the scalability of demonstrative oppositions occasions an empirical question: how do speakers employ demonstrative oppositions across scales? Much of the research on demonstrative use has investigated how speakers employ demonstrative oppositions in small-scale space, with targets in very close proximity to the deictic center. In contrast, we know little about the factors that influence demonstrative use when the search area for the target is at a larger scale, and when the target itself is likely to be larger.

### 2.3. Pointing and Scale

Pointing is the prototypical deictic gesture. Produced by extending an articulator to form or trace a line, a point invites the addressee to extend that line, conceptualizing a beam projected from the articulator and searching within that beam for an intended target (Kranstedt et al., 2006). Pointing is most often performed with the fingers, hand, and arm, and can take a variety of forms depending on how these articulators are configured to evoke a line (Kendon and Versante, 2003; Wilkins, 2003; Kendon, 2004; Hassemer and McCleary, 2018). Yet it is by no means limited to these articulators: a toss of the head, a jut of the chin, and/or funneling of the lips, combined with gaze in the target direction, are common indicating gestures in a variety of cultures (e.g., Sherzer, 1973; Enfield, 2001; Mihas, 2017) and may be preferred over manual pointing in some contexts (Cooperrider et al., 2018).

Pointing conveys information not only about the direction of the target, but also about its distance. Some research studies have found that pointing is more likely to occur when the target is farther away, so that its very presence suggests a relatively distant target (Bangerter, 2004; Cooperrider, 2011, 2015). Moreover, the form of the point itself conveys target distance via the *far-is-up* strategy—the farther the target, the higher the pointing arm. This strategy has been attested in pointing across a variety of cultures (Kendon and Versante, 2003; Wilkins, 2003; Mesh, 2017),(Mesh, submitted) and has even been found in non-human primates (Gonseth et al., 2017), suggesting that it may be a fundamental schema for representing distance.

Research on the factors influencing pointing—both its presence and its form—has largely focused on points toward manipulable objects relatively near the deictic center, and visually accessible to both members of the speech dyad (but for work in which visibility is manipulated, see Peeters and Özyürek, 2016). Exceptions to this trend have considered points toward targets in large-scale space without making a comparison between pointing strategies across multiple scales (cf. Mesh, 2017) (Mesh, submitted). As a consequence, we know little about whether pointing strategies shift as the scale of the search domain—and often the scale of the target itself—changes.

### 2.4. Co-organization of Multimodal Indicating Strategies and Scale

Demonstrative expressions and pointing can be produced and interpreted individually, but are much more often performed together (Diessel, 2006). The semantic contributions of each behavior are distinct, as not all of the perceptual and geophysical dimensions that they invoke are shared (Haviland, 2003, 2009; Kendon and Versante, 2003; Kendon, 2004). Yet the two indicating behaviors jointly facilitate the narrowing of the search domain (Levinson, 2003; Wilkins, 2003; Diessel, 2012). When they are co-produced, demonstratives and pointing are tightly temporally coordinated (Levelt et al., 1985; Chu and Hagoort, 2014; Krivokapic et al., 2016), suggesting that they are planned and organized together in speech production. They are also neurocognitively interpreted jointly (cf. Peeters et al., 2015a), providing further evidence for their connection.

Research on multimodal indicating is still in its early stages, yet the work to date has decisively shown that pointing and demonstratives are more than merely connected in function—they are manifestly co-organized (Bangerter, 2004; Cooperrider, 2011). Whether the two behaviors are coordinated in the same way for indicating at different scales, however, is still unknown.

### 2.5. Demonstratives and Pointing in Quiahije Chatino

#### 2.5.1. Setting: San Juan Quiahije, Oaxaca, Mexico

Quiahije Chatino is spoken by the ~3,600 inhabitants of the San Juan Quiahije municipality in Oaxaca, Mexico (INEGI, 2010). It is a variety of Eastern Chatino, one of three Chatino languages classified in the Zapotecan branch of the Otomanguean language stock (Campbell, 2013). The language is characterized by an intricate morphophonological system, with both grammatical and lexical distinctions encoded tonally (Cruz, 2011).

The Quiahije variety of Chatino is notably vital: children are still acquiring it as their first language, even as many of the surrounding Chatino communities are undergoing rapid language shift to Spanish (Cruz and Woodbury, 2014; Villard and Sullivant, 2016). Nevertheless, many of the Quiahije community's oral traditions are not being transmitted to younger generations (cf. Cruz, 2014). Recognizing that their community runs the risk of losing its traditions, community members in Quiahije have begun working with elders to preserve local knowledge. Early projects have focused on knowledge about the landscape and in particular on place names and practices for giving route directions (Cruz, 2017). Expertise in this domain was common in the community as recently as one generation ago, as community members navigated the mountainous terrain in the southern Sierra Madre mountain range to reach neighboring communities and to conduct trade. At present, there are many community elders who can faithfully describe the contours of trade routes that take as many as 5 days to walk (Smith Aguilar, 2017). For these speakers to locate crucial landmarks along the route, two linked indicating behaviors are indispensable: demonstrative expressions and pointing gestures.

#### 2.5.2. Demonstratives in Quiahije Chatino

Quiahije Chatino demonstratives are a closed and formally diverse class of five forms serving to indicate referents in relation to the deictic center. Four of the demonstrative forms are used for exophoric reference (i.e., reference to objects and entities in the real-world environment) and one form is used for discourse anaphoric reference. The exophoric demonstratives have been analyzed in terms of distance from and/or accessibility to the speech act participants (Cruz and Sullivant, 2012; Mesh, 2017). The preliminary analysis for the system is summarized in Table 1.

**Table 1:** The Quiahije Chatino demonstrative system.

**Demonstrative form(s)^**a**^**	**Gloss**	**Functional distinction**
re^C^/nde^C^	dem:1	Speaker-anchored proximal
kwa^J^	dem:2	Addressee-anchored proximal
kwa^F^	dem:n	Unmarked/neutral
kanq^G^	dem:d	Discourse anaphoric

a *We use a practical orthography to transcribe Chatino, rather than the International Phonetic Alphabet, and we represent the tone of each word using a superscripted letter. The orthography, including the letters assigned to each tone value, is presented in Appendix 1*.

Discourse-givenness and/or discourse focus appear to influence the choice of the speaker-anchored proximal forms, while other features of their semantics appear to be shared. As a consequence, we discuss “speaker-anchored forms” broadly in this paper.

All five demonstrative forms can occur as pronouns when preceded by the nominalizing particle *no*^*A*^, as shown in Example 2a^1^. All five forms can also occur as adnominals (adjectives) when preceded by a noun or followed by a relational noun, as shown in Example 2b. The exophoric demonstrative forms can occur as adverbs, alone or preceded by the locative particles *ti*^*H*^ or *ri*^*H*^, as shown in Example 2c.


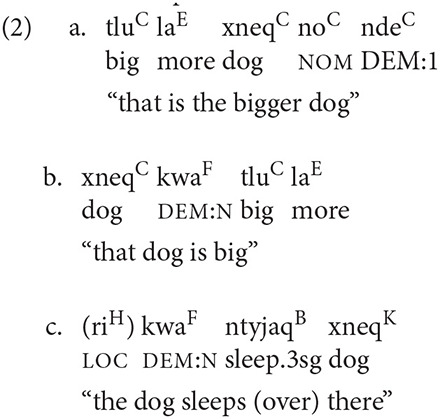


The current functional description of the Quiahije Chatino demonstrative system is based on elicited speaker judgments. No research to date has investigated demonstrative function and demonstrative choice in Quiahije Chatino speakers during spontaneous discourse.

#### 2.5.3. Pointing in San Juan Quiahije

Two forms of pointing are frequent in face-to-face interaction in Quiahije: the manual point and the chin point (a jut of the chin, optionally with pursed, extended lips). When using the hand and arm to point, Quiahije Chatino speakers have been shown to use the *far-is-up* strategy: the farther the target, the higher the pointing arm is raised^2^. Mesh (2017), (Mesh, submitted) analyzed video recorded interviews in which Quiahije Chatino speakers located landmarks near their homes and in the surrounding landscape, and found that speakers used the far-is-up strategy consistently when indicating targets with a distance range of 200 m to 107 km from the interview site. For this study, all targets were conceptualized as “at the landscape scale” and the notion of scale itself was not further explored. Chin pointing was not investigated, and to date there is no analysis of the contexts of use for chin pointing among Quiahije Chatino speakers.

## 3. Research Questions

Prior studies of demonstrative use and pointing in the Quiahije community have laid the groundwork for a more focused study of multimodal indicating in usage. Moreover, the central role of direction-giving in traditional community practices and the resurgence of interest in these practices through language revitalization projects in the community make such a study especially urgent.

For the current study, we pose the following research questions:

1. Does the distance of the indicated target influence:
  - the choice of demonstratives, across scales?
  - the presence of chin pointing, across scales?
  - the presence of manual pointing, across scales?
  - the form (height) of manual pointing, across scales?
2. Is there a relationship between demonstrative choice and use of pointing:
  - with the chin, across scales?
  - with the hand, across scales?

## 4. Current Study

### 4.1. Methods

#### 4.1.1. Participants

Data for the current study were drawn from interviews performed with eight native speakers of Quiahije Chatino (four female). Speakers were recruited by native speaker research assistants on the basis of their near-exclusive use of Chatino, though all participants showed at least some passive knowledge of Spanish (demonstrated outside of interviews, as participants heard questions posed by the first author in Spanish and responded in Chatino without waiting for interpretation). Interviews were performed in Quiahije Chatino by a native speaker of the language who is also fluent in Spanish, allowing for direct communication with the first author. Consent was obtained from all participants to use their research data, and many participants additionally gave permission to make their recorded image available to the public. Demographic information for all participants, including age, gender, languages used, and education level, is provided in Table 2.

**Table 2:** Participant information.

**Participant No**.	**Gender**	**Age**	**L1**	**L2**	**Education**
SJF13	F	67	Quiahije Chatino	Passive Spanish	None
SJF14	F	62	Quiahije Chatino	Passive Spanish	None
SJF15	F	63	Quiahije Chatino	Passive Spanish	None
SJF16	F	64	Quiahije Chatino	Passive Spanish	None
SJM11	M	56	Quiahije Chatino	Passive Spanish	Primary School
CM06	M	66	Quiahije Chatino	Passive Spanish	None
CM11	M	66	Quiahije Chatino	Passive Spanish	None
CM12	M	69	Quiahije Chatino	Spanish	Primary School

#### 4.1.2. Procedure

##### 4.1.2.1. Interview Design

Participants took part in an interview performed in six preselected stops along a 2.2-km walking trail to the peak of kyqya^C^ kcheq^B^ (“Thorn Mountain”), a location of religious and cultural significance to Chatino people in and outside of San Juan Quiahije. During the interview, participants discussed the role of six preselected stops on the trail in the annual religious pilgrimage performed by members of multiple Chatino communities. They also identified ten towns of importance in the surrounding district, and four towns vital to trade with communities in the larger state of Oaxaca. In keeping with our large-scale theme, targets prompted in the interviews involved named places. Such targets represent a class of sizeable and spatially stable entities of high sociocultural salience and interactional significance, as well as obvious relevance at the landscape scale (cf. Blythe et al., 2016). They were therefore deemed particularly suitable for our purposes. The locations of the interview stops, and the places to be discussed in each interview, were selected to elicit indicating behaviors with search domains at three scales:

- **Activity:** participants anticipated, and later reviewed, each of the six stops along the 2.2-km walking trail.
- **District:** participants discussed six towns at distances between 1.2 and 11 km from the walking trail.
- **State:** participants discussed four towns/cities in the state of Oaxaca, at distances between 16 and 108 km from the walking trail.

**Figure 1:**
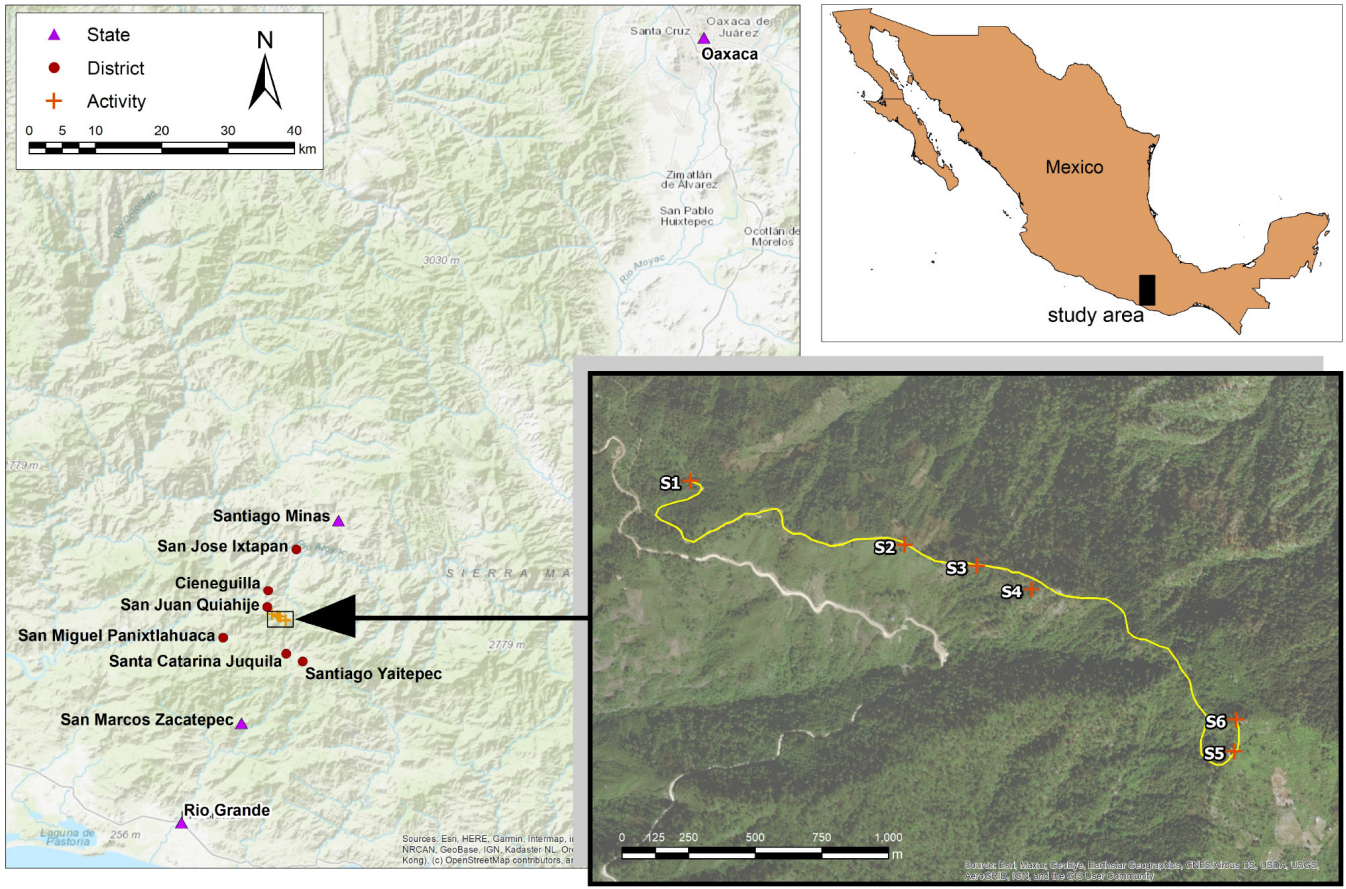
Targets discussed by interview participants at six different locations along the trail.

Figure 1 presents the trail with the full set of 16 targets. Targets at the activity, district, and state scales are distinguished by the color and style of their placemarks. Each of the search domain scales can be defined as a span of distance from the speech dyad (i.e., the interviewer and participant). The scales can also be distinguished by the general characteristics of the search domains, as presented in Table 3.

**Table 3:** Scales for search domains, with their characteristics.

**Scale**	**Distance (km)**	**General characteristics**
Activity	<2.2	Area of ongoing interview activity, within the speech dyad and beyond
District	1.2–11	Local area of Chatino residence/identity
State	>11	Regional area of Chatino identity/trade

All 16 targets were discussed at each stop, as participants (in the role of senders) were prompted to provide to the interviewer (in the role of addressee): (1) the name of the current trail stop and its origins, (2) the names of the towns visible from the stop and their origins, and (3) the rough direction of all targets (minus the current stop), as gauged from the current stop. The full interview protocol, including the walk to the peak of kyqya^C^ kcheq^B^ (but excluding the subsequent descent) required a total of 3 h, ~60 min of which were spent performing interviews at the preselected stops along the route. The full interview protocol has been made available with the Supplementary Material for this paper.

##### 4.1.2.2. Recording Procedure

All interviews were video recorded from two perspectives (giving front and side views of the participants) using Garmin Virb action cameras. The interviewer and participant each wore a head-mounted Røde HS2 headset microphone connected to a Røde Wireless Go transmitter with a receiver that attached directly to one of the action cameras. Digital video was recorded by the first author and a trained research assistant, neither of whom participated in the interview. Video was shot in MP4 format with a video mode of 1080p and a frame rate of 30 fps. The Virb action cameras additionally collected geoinformation, producing a GPX file containing the coordinates of each camera (collected at a rate of 10 Hz).

#### 4.1.3. Dataset

Since each participant was recorded during interviews at six locations along the trail, the dataset consisted of a total of 48 video recordings. The recordings ranged in length from 2:59 to 8:24 min (*M* = 5:34 *SD* = 1:14). We excluded recordings from the first trail stop, treating the interviews recorded there as a training activity in which participants were familiarized with the task of indicating 16 targets. This left a dataset of 42 recordings for analysis.

#### 4.1.4. Data Treatment and Coding

The audio tracks from both cameras were combined to produce a single integrated sound file in WAV format, and the video recording start times were synched, using Adobe Premier. The digital video and audio files were transcribed, translated and coded using frame-by-frame analysis, performed in the video annotation software, ELAN (2020).

For this study, the unit of analysis was the *indicating act*, defined as all behaviors—spoken and gestural—that occurred during a stretch of speech in which a demonstrative expression was used, ±1 s. Speech was used as the point of entry to the data: demonstrative expressions in all three formal contexts (pronominal, adnominal, adverbial) were identified, and the prosodic units in which they occurred were assigned to indicating acts. By definition, then, all indicating acts contained at least one demonstrative expression. An indicating act could additionally contain one or multiple pointing gestures, produced by jutting the chin or extending a hand/arm.

##### 4.1.4.1. Speech

*4.1.4.1.1. Transcription*. Three research assistants (native speakers of Quiahije Chatino) with experience writing the language watched the video recorded interviews and identified every reference to our pre-selected targets. They identified the first three cases where a demonstrative expression was used to indicate each target. They then identified the *breath unit* surrounding each demonstrative expression, defined as the stretch of phonation visible on a waveform viewer, bounded on both sides by a lack of phonation (cf. Lieberman, 1967). They transcribed all talk in each breath unit, following the orthographic conventions of Cruz (2011), and produced a corresponding translation to Spanish (the language of communication between the research assistants and the first author).

*4.1.4.1.2. Speech coding*. Each indicating act was coded for the demonstrative form it contained. For those indicating acts that encompassed multiple demonstratives with the same target, only the first demonstrative was coded, to preserve the independence of the data points. If an indicating act contained two demonstratives with different targets, e.g., “Sour rock is *here* and Turkey Breast Rock is *there*,” the speech was reanalyzed into two separate indicating acts, and each was assigned a code for demonstrative form. This resulted in a set of 883 indicating acts in total.

##### 4.1.4.2. Gestures

*4.1.4.2.1. Gesture identification*. All gesture coding was performed with the audio switched off, and with transcriptions and translations hidden. This ensured that coders had no access to the content of the speech in the recordings.

Gestures with strokes that occurred inside the boundaries of an indicating act were identified, and assigned to the corresponding indicating act. To do this, the first author proceeded frame-by-frame, first identifying all manual gesture units (from the onset of a spatial excursion of the fingers, hand and/or arm to the assumption of a rest position) as well as head movements that might constitute a deictic chin point. The first author then identified the stroke phase within each of the identified manual gesture units. The boundaries of the stroke were identified via changes in the velocity of the hand movement (such as when the movement slowed, or stopped altogether in the case of static strokes) and/or changes in the handshape (cf. Kendon, 1972; Kita et al., 1998; Seyfeddinipur, 2006).

When no stroke boundary could be identified using these criteria, the stroke was identified in the frame(s) in which the articulators (fingers, hand, and/or arm) were at the point of fullest extension. Self-regulators (gestures touching body or face, cf. Ekman and Friesen, 1969) and gestures with a possible “pragmatic” function (such as conveying the speaker's epistemic stance toward their statement, often diagnosed via palm-up gesture forms, cf. Kendon, 2004; Müller, 2004) were excluded from analysis^3^.

*4.1.4.2.2. Gesture coding*. Each gesture contained within an indicating act was coded as C (chin point), M (manual point), or CM (chin point and manual point). If multiple manual gestures or multiple chin points occurred within a single indicating act, the first token of each gesture type was coded. Of the 882 indicating acts identified, 68 contained a chin point, 416 contained a manual point, and 8 contained both pointing types.

One formational feature of the manual points was further coded: the elbow height of the arm during the articulation of each stroke was coded as low (below shoulder) or high (at or above shoulder). A first coder coded elbow height in the full dataset, while a second coder, assigning height values to a set of pre-identified strokes, coded one randomly selected video recording from each participant (~17% of the dataset). We computed inter-rater reliability measures (Hallgren, 2012) using R version 3.6.1 (R Core Team, 2019) with the irr package (Gamer et al., 2019), and found that the two coders showed agreement in 93% of cases (Cohen's kappa = 0.85).

##### 4.1.4.3. Target Distance

Each indicating act was assigned a distance measure, reflecting the geodesic distance in meters between the interview site and the target location. Geodata (latitude–longitude pairs stored in Garmin's proprietary GMetrix file format) were extracted from a single interview, and one representative latitude–longitude pair was identified at the approximate center of each of the interview stops along the trail. A latitude–longitude pair was also identified at the approximate center of each of the off-trail targets, allowing for the distance between the interview location and target location to be measured in a geographic information system (GIS).

Table 4 presents distance measures for each target discussed in the interviews. Targets are identified by their Chatino names, as well as by conventional placenames assigned by Spanish speakers (or, in the absence of these conventions, by a translation from Chatino to English). The reported distance range represents the minimum and maximum possible distances between the target and the stops along the trail.

**Table 4:** Walking interview targets: scale, names, and distance range.

**Scale**	**Name, Quiahije Chatino**	**Name, Translated**	**Distance (km)**
Activity	ntenq^F^ tiyuq^G^	Plain of the Spring	0–2.2
	tu^C^ kchi^C^	Mountain Pass	0–1.4
	ke^A^ tiyeq^B^	Sour Rock	0–1.2
	ke^A^ ku^E^ suq^C^	Turkey Breast Rock	0–1.3
	lo^A^ si^K^ kyqya^C^ kcheq^B^	Petition Monument	0–2.2
	kyqya^C^ kcheq^B^	Peak of Thorn Mountain	0–2.2
District	kchin^A^	San Juan Quiahije	1.3–3.4
	ntenq^F^	Cieneguilla	3.6–5.2
	tqwa^A^ tyku^E^	San José Ixtapan	10.4–11.0
	skwi^E^	San Miguel Panixtlahuaca	7.9–9.4
	sqwe^F^	Santa Catarina Juquila	4.9–6.3
	ke^G^ xin^E^	Santiago Yaitepec	6.6–8.4
State	se^A^ na^A^ nya^K^	Santiago Minas	16.8–17.1
	tsi^C^	San Marcos Zacatepec	16.7–1.07
	ntenq^F^ tyku^E^ jlyu^B^	Rio Grande	33.9–34.2
	lo^A^ ntqa^B^	Oaxaca City	106.6–107.0

##### 4.1.4.4. Data Exclusion

A total of 283 coded indicating acts were removed from the dataset for the following reasons. Indicating acts containing the discourse-anaphoric demonstrative *kanq*^*G*^ (*n* = 120) were excluded in order to narrow the dataset to cases of exophoric reference (i.e., reference to concrete, physical entities in the space around the speech dyad). Indicating acts containing the addressee-anchored proximal forms *kwa*^*J*^ and *kwa*^*E*^ (*n* = 24) were removed because their infrequent occurrence did not support a statistical analysis. Indicating acts containing demonstratives with multiple targets (e.g., “Sour Rock and Turkey Breast Rock are *there*”) could not be assigned a single measure for target distance. This was also the case for indicating acts in which a single pointing gesture had multiple targets (because it accompanied a demonstrative expression with multiple targets, or because it extended across multiple demonstrative expressions with discrete targets). All such indicating acts (*n* = 96) were excluded. Indicating acts that contained speech with segment-by-segment route directions (*n* = 38) were excluded, since in these cases speakers often used demonstratives in set phrases roughly equivalent to *from there we go on*, and it is unclear whether these cases are comparable to other indicating speech. Indicating acts containing two pointing types (*n* = 4) were excluded to simplify the analysis.

After these exclusions, the dataset for analysis comprised a total of 601 indicating acts, all with an exophoric function. Of these, all contained a demonstrative, 35 contained an additional chin point, and 256 contained an additional manual point. Notably, after exclusions the dataset contained only three demonstrative forms: the speaker-anchored proximal forms *nde*^*C*^ or *re*^*C*^, which we treat jointly in our analysis, and the unmarked/neutral form *kwa*^*F*^. We hereafter refer to these forms as the “speaker-proximal” and “neutral” forms.

#### 4.1.5. Data Analysis

The goal of the analysis was to test whether target distance influenced how multimodal indicating was performed within and across three scales—activity, district, and state.

We treated distance in two ways for our analyses. For descriptive tables, we subdivided the distance range within each scale into bins. For the activity scale, we created four bins of 0–541, 542–1,082, 1,083–1,623, and 1,624–2,206 m. For the district scale, we created four bins of 0–2,751, 2,752–5,502, 5,503–8,253, and 826–11,004 m. For the state scale, we created three bins spanning the distances where our targets clustered, with spans of 0–19,000, 19,001–36,000, and 36,001–108,000 m). This treatment of distance as categorical allowed us to present descriptive statistics in terms of the distribution of demonstrative forms and pointing use across distance categories.

For statistical analyses, we took a different approach. Within each scale, the actual distance values (in m) were rescaled to values from 0 (i.e., the minimal distance within the scale) to 1 (i.e., the maximal distance within the scale). This transformation leaves the relative differences between the values within each scale intact. At the same time, it facilitates the comparison of the distance effects across the three scales because the estimated effects (regression coefficients) receive an equivalent interpretation (i.e., whether the change in occurrence of the outcome variable at the maximal distance within a scale compared with that at the minimal distance within a scale).

We performed six separate statistical analyses to answer the research questions. In the first four analyses, we looked at the effects of distance and scale on choice of demonstrative form (speaker-proximal or neutral), the presence of a chin point, the presence of a manual point, and the height of a manual point (low or high). Since we wanted to know whether the effect of distance varied across scales, we primarily looked at the interaction of these two predictors. If this interaction was significant, we tested the individual effects of distance within the activity, district, and state scales (simple main effects). If the interaction effect was not significant, it was removed from the analysis model, to see whether any of the remaining effects were significant.

In the final two analyses, we looked at the effect of choice of demonstrative on the presence of a chin point and on the presence of a manual point, again within each of the three scales. The procedure for testing these two effects was similar as the one for the first four analyses: We primarily looked at the interaction of choice of demonstrative and scale. If this interaction was significant, then we looked at the simple main effects of demonstrative within each of the three scales. Otherwise, we removed it from the analysis to see whether any of the other remaining effects was significant.

All six analyses were mixed effects logistic regression models with scale and distance (analyses 1–4) or scale and choice of demonstrative (analyses 5 and 6) as fixed factors, and participant as a random factor. In the results section, we provide estimates (*EST*), standard errors (*SE*), *z*-values, and *p*-values for the effects that are most relevant for the research questions. All *p*-values for simple main effects have been corrected for multiple comparisons (Dunnett's method). A list of the six regression models (fixed effects parts only) is given in Appendix 3. The analysis was performed in R version 3.6.1 (R Core Team, 2019) using the packages lme4 (Bates et al., 2015) and multcomp (Hothorn et al., 2008).

### 4.2. Results

#### 4.2.1. Sample Description

The dataset consisted of 601 indicating acts, of which 235 had targets at the Activity scale, 238 had targets at the District scale, and 128 had targets at the state scale. Every indicating act contained one demonstrative expression—using a speaker-proximal form (*re*^*C*^ or *nde*^*C*^) or a neutral form (*kwa*^*F*^)—while 35 contained an additional chin point, and 255 contained an additional manual point.

Across participants, manual points and the two demonstrative forms were used to refer to all 16 targets, but chin points were used to refer to 11 targets only. Across targets, all participants used the two demonstrative forms and every participant used manual points at least six times and chin points at least twice. There was natural variation across targets and participants in the frequencies of demonstratives and indicating strategies. For example, manual points comprised 95% of the pointing gestures of some participants (with chin points accounting for the other 5%), while for other participants, manual points comprised 60% of their pointing gestures (with chin points accounting for the remaining 40%). The distribution of indicating strategies and indicating forms, across targets and across participants, is presented in Appendix 2.

#### 4.2.2. Effect of Distance on Demonstrative Choice

Our research question (1a) asked whether the distance of the indicated target influences demonstrative choice, and whether this effect is found across multiple scales. The distribution of demonstrative forms across distance categories is presented in Table 5. This distribution suggested that participants were more likely to use a speaker-proximal demonstrative form when the target was closer to the speech dyad, but only for targets at the activity scale. The interaction between distance and scale was significant (see the Appendix 3.1), and subsequently pursued by an analysis of simple main effects (i.e., the effect of distance within each level of scale). A significant simple main effect of distance was found only in the activity scale: participants were more likely to use the speaker-proximal form when the target was relatively near to them on the trail and less likely to use this form as the distance to the activity scale targets increased (*EST* = 2.832, *SE* = 0.520, *z* = 5.452, *p* = 0.000). No effects were found at the district scale (*EST* = 0.611, *SE* = 0.476, *z* = 1.282, *p* = 0.488) or at the state scale (*EST* = 0.255, *SE* = 0.406, *z* = 0.629, *p* = 0.896).

**Table 5:** Raw frequencies (with proportions in parentheses) of demonstrative forms across distance categories at the activity, district, and state scales.

**Dist. Cat**.	**Activity**				**District**				**State**			
	**re**^**C**^**/nde**^**C**^		**kwa**^**F**^		**re**^**C**^**/nde**^**C**^		**kwa**^**F**^		**re**^**C**^**/nde**^**C**^		**kwa**^**F**^	
1	117	(0.85)	21	(0.15)	12	(0.75)	4	(0.25)	34	(0.54)	29	(0.46)
2	21	(0.46)	25	(0.54)	43	(0.61)	27	(0.39)	9	(0.38)	15	(0.62)
3	11	(0.35)	20	(0.65)	36	(0.59)	25	(0.41)	19	(0.46)	22	(0.54)
4	10	(0.50)	10	(0.50)	53	(0.58)	38	(0.42)				
SUM	159	(0.68)	76	(0.32)	144	(0.61)	94	(0.39)	62	(0.48)	66	(0.52)

#### 4.2.3. Effect of Distance on the Use of Chin Pointing

Our research question (1b) asked whether the distance of the indicated target influences the use of chin pointing, and whether this effect is found across multiple scales. The distribution of chin pointing (vs. its absence) across distance categories is presented in Table 6. The descriptive results reflect the relatively small number of chin points produced in the study: only 35 in total. With such a small number of cases, we would be unlikely to find a strong relationship between the distance of the target and the use of chin pointing. The results of the analysis showed no significant joint effect of distance and scale, nor any significant effect of distance or scale when used as individual predictors (see Appendix 3.2).

**Table 6:** Raw frequencies (with proportions in parentheses) of chin points at the activity, district, and state scales.

**Dist. Cat**.	**Activity**				**District**				**State**			
	**Chin pt**.		**No**		**Chin pt**.		**No**		**Chin pt**.		**No**	
1	7	(0.05)	131	(0.95)	2	(0.13)	14	(0.87)	0	(0.00)	63	(1.0)
2	1	(0.02)	45	(0.98)	5	(0.07)	65	(0.93)	4	(0.16)	20	(0.84)
3	4	(0.12)	27	(0.78)	4	(0.07)	57	(0.93)	3	(0.07)	38	(0.93)
4	1	(0.05)	19	(0.95)	4	(0.04)	87	(0.96)				
SUM	13	(0.05)	222	(0.95)	15	(0.07)	223	(0.93)	7	(0.05)	121	(0.95)

#### 4.2.4. Effect of Distance on the Use of Manual Pointing

Our research question (1c) asked whether the distance of the indicated target influences the use of manual pointing, and whether this effect is found across multiple scales. The distribution of manual pointing (vs. its absence) across distance categories is presented in Table 7. The descriptive results suggested that the distance of the target did not influence whether participants pointed at the district and state scales. Only in the activity scale did the descriptive data suggest an effect of distance: here it appeared that participants were more likely to use a manual point when the target was farther from the speech dyad. The interaction between distance and scale was significant (see the Appendix 3.3), and subsequently pursued by an analysis of simple main effects (i.e., the effect of distance within each level of scale). The analysis showed a significant main effect of distance only for the activity scale: participants were least likely to use the manual point when the target was nearest to the deictic center, and more likely to point with the hand as the distance to the activity scale targets increased (*EST* = 1.606, *SE* = 0.487, *z* = 3.296, *p* = 0.000). No effects were found at the district scale (*EST* = −0.205, *SE* = 0.478, *z* = 0.428, *p* = 0.964) or at the state scale (*EST* = 0.157, *SE* = 0.418, *z* = 0.374, *p* = 0.975).

**Table 7:** Raw frequencies (proportion) of manual points at the activity, district, and state scales.

**Dist. Cat**.	**Activity**				**District**				**State**			
	**Manual pt**.		**No**		**Manual pt**.		**No**		**Manual pt**.		**No**	
1	30	(0.22)	108	(0.78)	8	(0.50)	8	(0.50)	33	(0.52)	30	(0.48)
2	18	(0.39)	28	(0.61)	41	(0.59)	29	(0.41)	11	(0.46)	13	(0.54)
3	12	(0.39)	19	(0.61)	32	(0.52)	29	(0.48)	22	(0.54)	19	(0.46)
4	9	(0.45)	11	(0.55)	40	(0.44)	51	(0.56)	0	(0.00)		
SUM	69	(0.29)	166	(0.71)	121	(0.51)	117	(0.49)	66	(0.51)	62	(0.49)

#### 4.2.5. Effect of Distance on the Elbow Height of Manual Points

Our research question (1d) asked whether the distance of the indicated target influences the form (height) of manual pointing, and whether this effect is found across multiple scales. The height values of manual pointing across distance categories are presented in Table 8. The descriptive results suggested that distance weakly influenced pointing height at the activity scale alone. The interaction between scale and height was significant (see Appendix 3.4) and pursued with an analysis of simple main effects. We found a marginally significant effect of distance within the activity scale: as targets increased in distance, participants were more likely to raise the elbow of the pointing arm at the activity scale (*EST* = 1.985, *SE* = 0.907, *z* = 2.187, *p* = 0.084). No effects were found at the district scale (*EST* = 0.213, *SE* = 0.713, *z* = 0.299, *p* = 0.987) or state scale (*EST* = −0.420, *SE* = 0.676, *z* = −0.621, *p* = 0.899).

**Table 8:** Raw frequencies (proportion) of elbow height values for manual points at the activity, district, and state scales.

**Dist. Cat**.	**Activity**				**District**				**State**			
	**Low**		**High**		**Low**		**High**		**Low**		**High**	
1	21	(0.70)	9	(0.30)	4	(0.50)	4	(0.50)	8	(0.24)	25	(0.76)
2	11	(0.61)	7	(0.39)	14	(0.50)	24	(0.50)	1	(0.09)	10	(0.91)
3	5	(0.42)	7	(0.58)	12	(0.38)	20	(0.62)	7	(0.32)	15	(0.68)
4	3	(0.33)	6	(0.67)	16	(0.42)	22	(0.58)				
SUM	40	(0.58)	29	(0.42)	46	(0.40)	70	(0.60)	16	(0.24)	50	(0.76)

Notably, the height of manual points appeared to shift between the scales, with low elbow predominating at the activity scale, and a high elbow at the district and state scales. To test this observation, we simplified the logistic regression model, using scale alone as a fixed factor and participant as a random factor. We found a significant main effect of scale: participants were more likely to point with a raised arm to targets at the district scale (*EST* = 0.835, *SE* = 0.338, *z* = 2.468, *p* = 0.117) and at the state scale (*EST* = 1.784, *SE* = 0.431, *z* = 4.144, *p* = 0.000), compared to the activity scale.

#### 4.2.6. Relationship Between Demonstrative Form and Use of Chin Pointing

Our research question (2a) asked whether there is a relationship between demonstrative choice and use of pointing with the chin, and whether this effect is found across multiple scales. The distribution of chin pointing and demonstrative choice across distance categories is presented in Table 9. With just 35 observations of chin points in the dataset, we did not anticipate an analysis to reveal a strong relationship between the use of chin pointing and the choice of a speaker-proximal or distal demonstrative. The analysis showed no significant interaction between choice of demonstrative and scale (see Appendix 3.5).

**Table 9:** Raw frequencies (proportion) of demonstrative forms and chin points at the activity, district, and state scales.

**Dem. choice**	**Activity**				**District**				**State**			
	**Chin pt**.		**No**		**Chin pt**.		**No**		**Chin pt**.		**No**	
*nde^C^/re^C^*	6	(0.04)	153	(0.96)	6	(0.04)	138	(0.96)	4	(0.06)	58	(0.94)
*kwa^F^*	7	(0.09)	69	(0.91)	9	(0.10)	85	(0.90)	3	(0.05)	63	(0.95)
SUM	13	(0.06)	222	(0.94)	15	(0.06)	223	(0.94)	7	(0.05)	121	(0.95)

#### 4.2.7. Relationship Between Demonstrative Form and Use of Manual Pointing

Our research question (2b) asked whether there is a relationship between demonstrative choice and use of pointing with the hand, and whether this effect is found across multiple scales. The distribution of manual pointing and demonstrative choice across distance categories is presented in Table 10. The descriptive results suggested a relationship between the use of a manual point and the use of a speaker-proximal demonstrative form. The interaction between choice of demonstrative and scale was marginally significant (see Appendix 3.6). In addition, the removal of the interaction did not result in a significantly worse model (χ^2^ = 3.549, *df* = 2, *p* = 0.170). In this model without the interaction, both scale and demonstrative form showed a significant relationship with manual points. There were overall more manual points with *nde*^*C*^*/re*^*C*^ than with *kwa*^*F*^ (*EST* = −0.873, *SE* = 0.195, *z* = −4.469, *p* = 0.000), and, compared to the activity scale, this effect was stronger at the district scale (*EST* = 1.099, *SE* = 0.208, *z* = 5.262, *p* = 0.000) and at the state scale (*EST* = 1.262, *SE* = 0.251, *z* = 5.024, *p* = 0.000), compared to the activity scale.

**Table 10:** Raw frequencies (proportion) of demonstrative forms and manual points at the activity, district, and state scales.

**Dem. choice**	**Activity**				**District**				**State**			
	**Manual pt**.		**No**		**Manual pt**.		**No**		**Manual pt**.		**No**	
*nde^C^/re^C^*	50	(0.31)	109	(0.69)	82	(0.57)	62	(0.43)	41	(0.66)	21	(0.34)
*kwa^F^*	19	(0.25)	57	(0.75)	39	(0.41)	55	(0.59)	25	(0.38)	41	(0.62)
SUM	69	(0.29)	166	(0.71)	121	(0.51)	117	(0.49)	66	(0.52)	62	(0.48)

### 4.3. Discussion

#### 4.3.1. Target Distance Influences Demonstrative Choice and Manual Pointing, Only in Activity Scale Space

For this study, we defined three scales for the search domains of indicating acts. The scales differed in their spatial extent and in other characteristics, as described in Table 3. We asked whether the factor of target distance would have an effect on multimodal indicating behaviors, and, if the effect were present, whether it would be the same across the three scales.

A prominent finding from this study is that distance had an effect on indicating behaviors at only one scale. The activity scale—the smallest scale in the study design—was the one at which participants showed sensitivity to target distance, both in their demonstrative choice and in their use and modulation of manual pointing.

##### 4.3.1.1. Demonstrative Choice

Participants were significantly more likely to use a speaker-proximal demonstrative when activity scale targets were near them. As the distance to the target increased, so did the likelihood that participants would use the neutral demonstrative form. At the district and state scales, by contrast, the speaker-proximal and neutral forms were used with near-equal frequency: there was no significant effect of distance on the choice of demonstrative forms. We illustrate these findings below, with examples from the video data.

In Example 3, a participant stands at Petition Monument, the fifth stop on the kyqya^C^ kcheq^B^ trail. The final stop on the trail lies 120 m ahead, through a wooded path. The participant uses the speaker-proximal demonstrative to indicate the stop (3a). Later, the participant explains that she and the interviewer stopped at every landmark on the trail, and indicates the farthest one with the neutral demonstrative (3b).


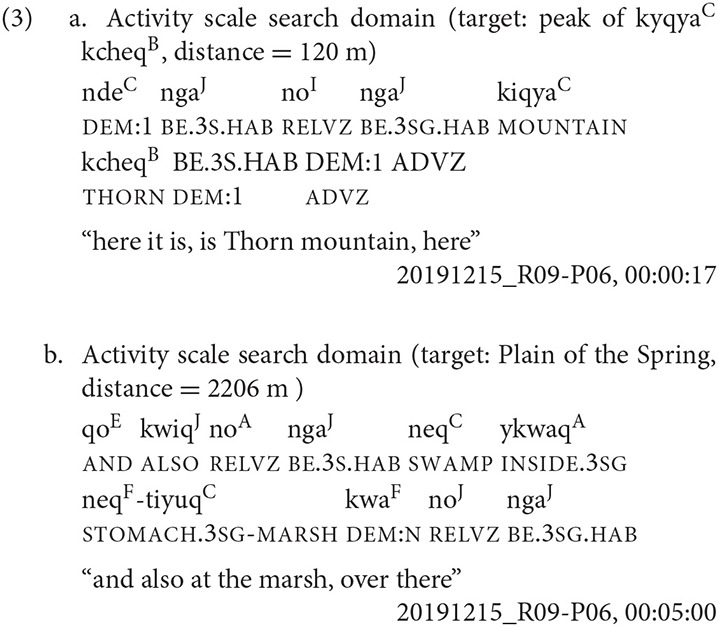


In considering the study results for demonstrative choice, we take note of the contrast between the activity scale and the two larger scales operationalized for the study. We observe that our participants showed sensitivity to target distance in activity scale space in much the same way as participants in a variety of experimental studies have done when indicating targets in “interaction-scale” or tabletop space. We interpret this as evidence that the demonstrative oppositions long explored in smaller-scale space *can* scale up—though not without limit. Our results suggest that distance effects on oppositions at smaller scales disappear at larger ones.

It is noteworthy that the point at which the distance effect disappears in our study coincides with the outer perimeter of the hiking activity itself. This suggests that the participants' conception of the target as co-present with both sender and addressee in a shared activity space is central to the use of demonstrative oppositions—an explanation that has been favored in accounts of social factors driving demonstrative choice (especially Enfield, 2003, 2018). Our results at the activity scale suggest that distance exerts some influence on demonstrative choice, though this influence may well be conditioned, or even eclipsed, by other social-pragmatic factors.

##### 4.3.1.2. Manual Pointing: Presence and Form

Distance influenced two aspects of manual pointing to targets at the activity scale. First, the distance of the target influenced the *presence* of a manual point: participants were more likely to point to targets using their hand when targets were farther from them. In addition, distance had a marginal influence on the *form* of the manual point: for targets farther away, participants were more likely to raise the pointing arm until the elbow was at the level of the shoulder or above.

**Figure 2:**
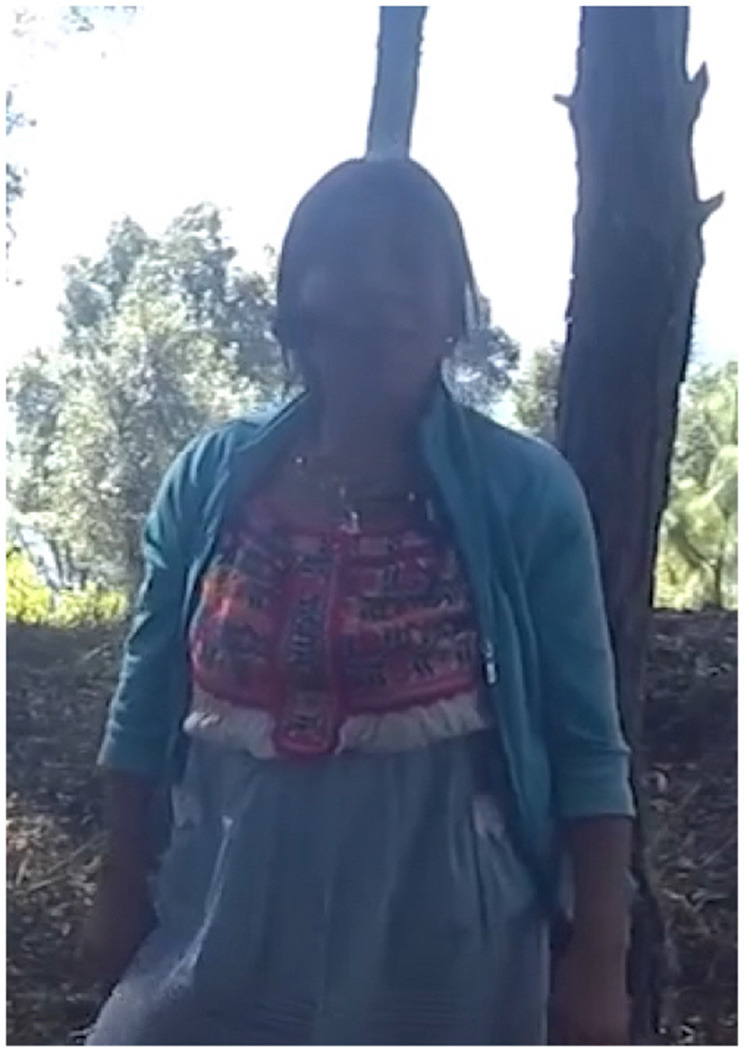
Activity scale [target: Peak of kyqya^C^ kcheq^B^].

**Figure 3:**
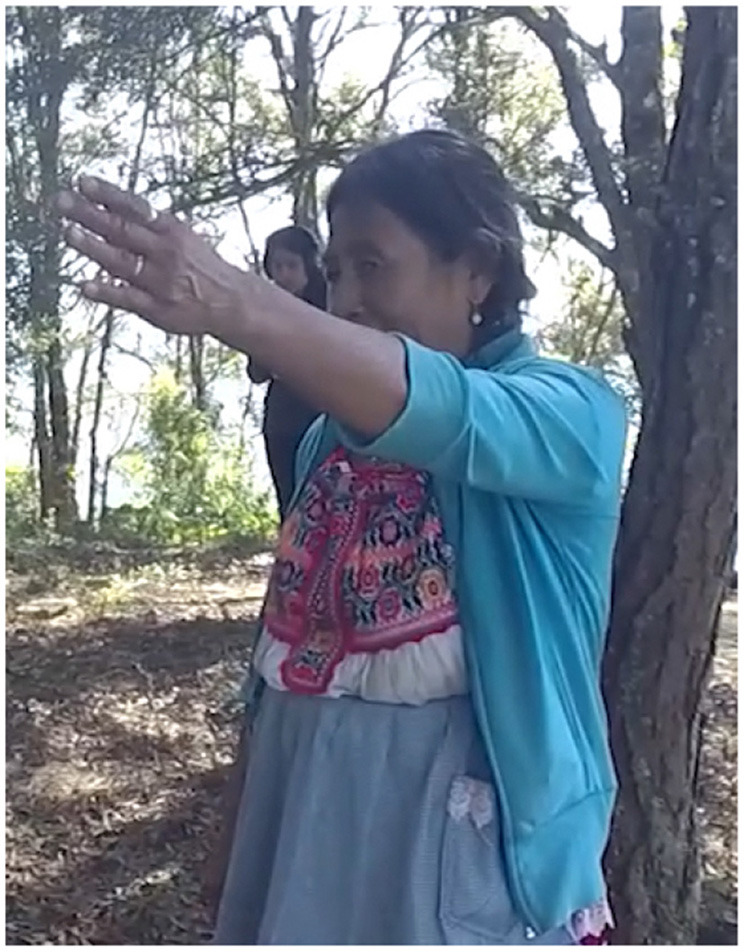
District scale [target: ke^A^ ku^E^ suq^C^].

In Example 4, the participant stands at the Petition Monument, the fifth stop on the kyqya^C^ kcheq^B^ trail. She indicates the nearest trail stop using a low elbow alongside the speaker-proximal demonstrative, *nde*^*C*^ (Example 5, Figure 2). When describing the stop at Turkey Breast Rock 950 m away, she indicates the more distant location using a pointing gesture with a high elbow, alongside the neutral demonstrative *kwa*^*F*^ (Example 4b, Figure 3). Notably, this distant target is at a lower altitude than the speaker, making her pointing form interpretable only as an application of the far-is-up schema for encoding distance^4^.

[Fn fn0005]
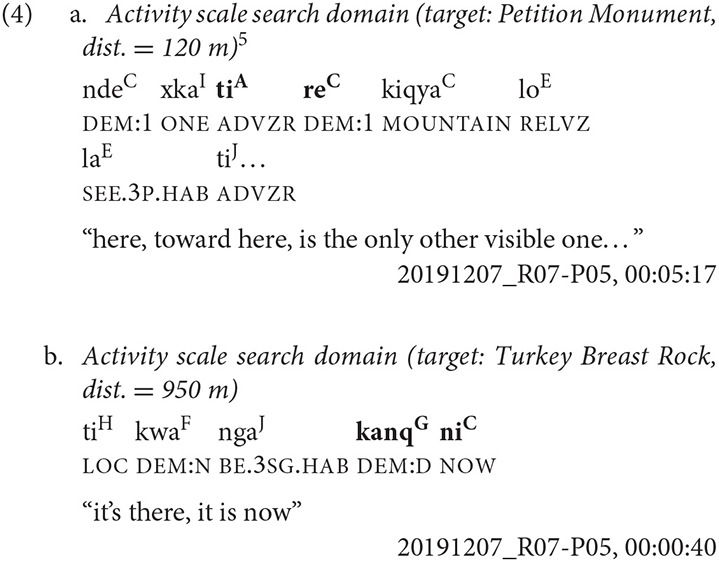


Here again, we see a pattern at our smallest distance scale that parallels observed patterns in “interaction-scale” space. In the laboratory and on the trail, participants are more likely to point to targets when they are farther away (cf. Bangerter, 2004; Cooperrider, 2015), and show sensitivity to the distance of the target by modulating the form of the point itself. Again, this sensitivity appears to be bounded. Neither the presence nor the form of a manual point appears to be influenced by distance beyond what, for our study, amounts to activity scale space.

#### 4.3.2. Pointing Form Is a Cue to Scale Itself

There was also a strong effect of scale itself on the height of the pointing gestures. For targets at the activity scale, distance did prompt raising of the pointing arm, yet participants were more likely overall to point with a lowered arm. For district scale targets, participants were more likely to point with a raised arm, and likelier still to do so when indicating state scale targets. These findings are illustrated in the examples that follow.

**Figure 4:**
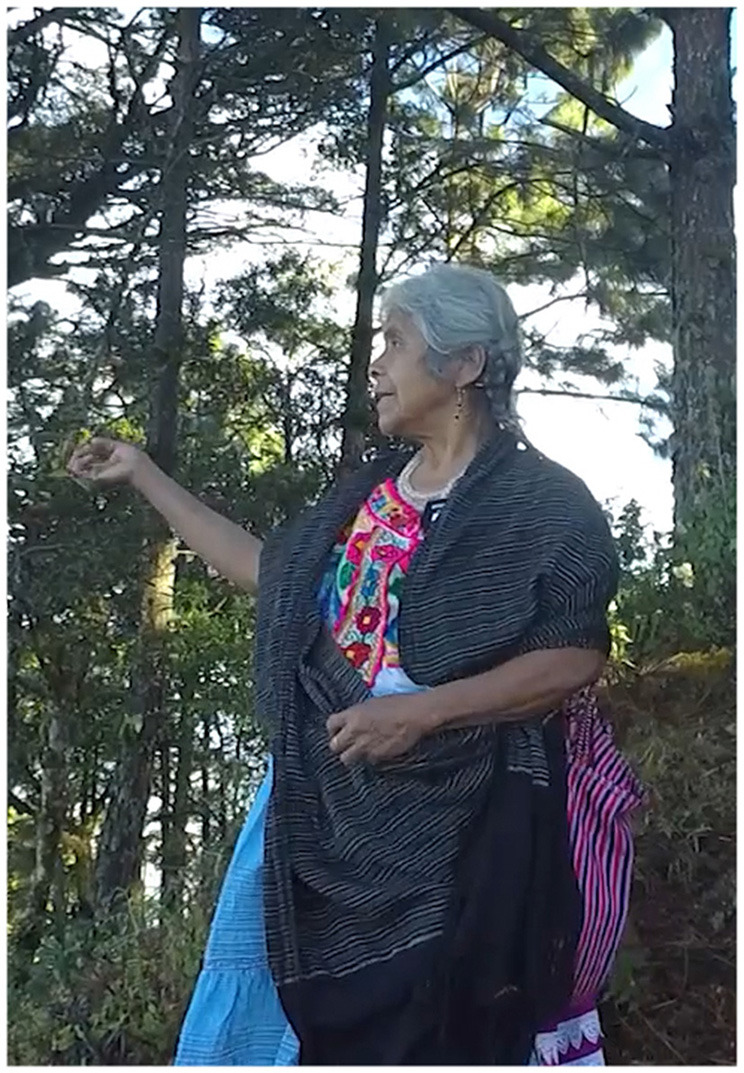
Activity scale [target: Plain of the Spring].

**Figure 5:**
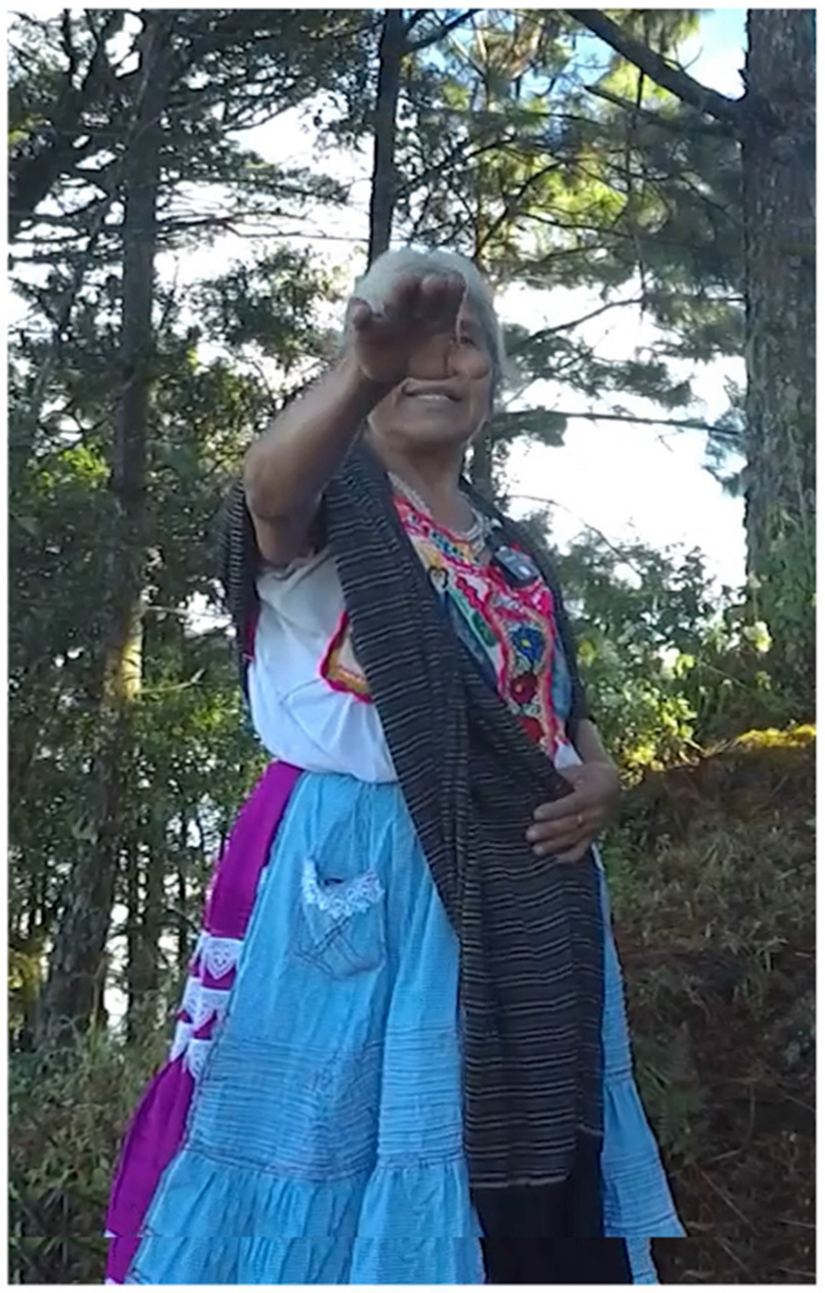
District scale [target: Santa Catarina Juquila].

**Figure 6:**
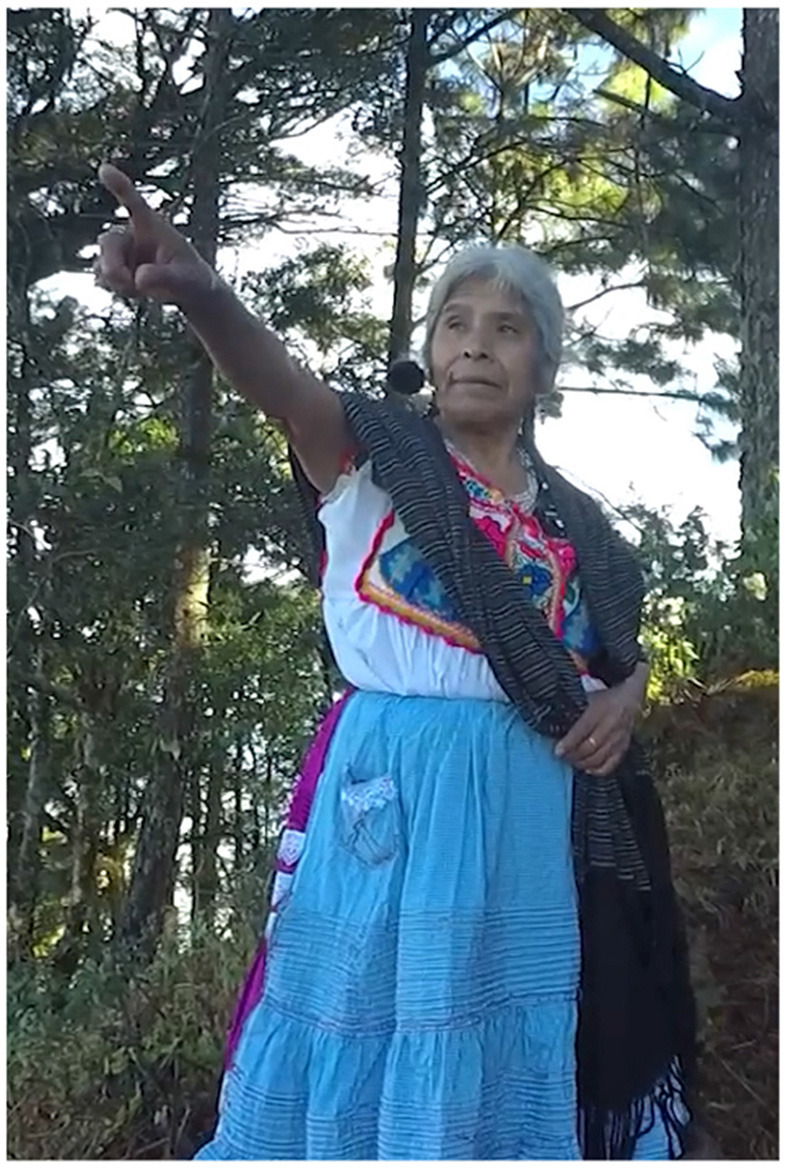
State scale [target: Rio Grande].

In Example 5, the participant is standing at Sour Rock, the third stop on the kyqya^C^ kcheq^B^ trail. When asked about Plain of the Spring, an activity scale target, she indicates its location using the speaker-proximal demonstrative *nde*^*C*^ and a manual point with a low elbow (Example 5a, Figure 4). To locate Santa Catarina Juquila, a district scale target, she indicates the town using the proximal demonstrative *nde*^*C*^ and a point with a raised elbow (Example 5b, Figure 5). When indicating Rio Grande, a state scale target, she uses the neutral demonstrative *kwa*^*F*^ and a point with a raised elbow (Example 5c, Figure 6).


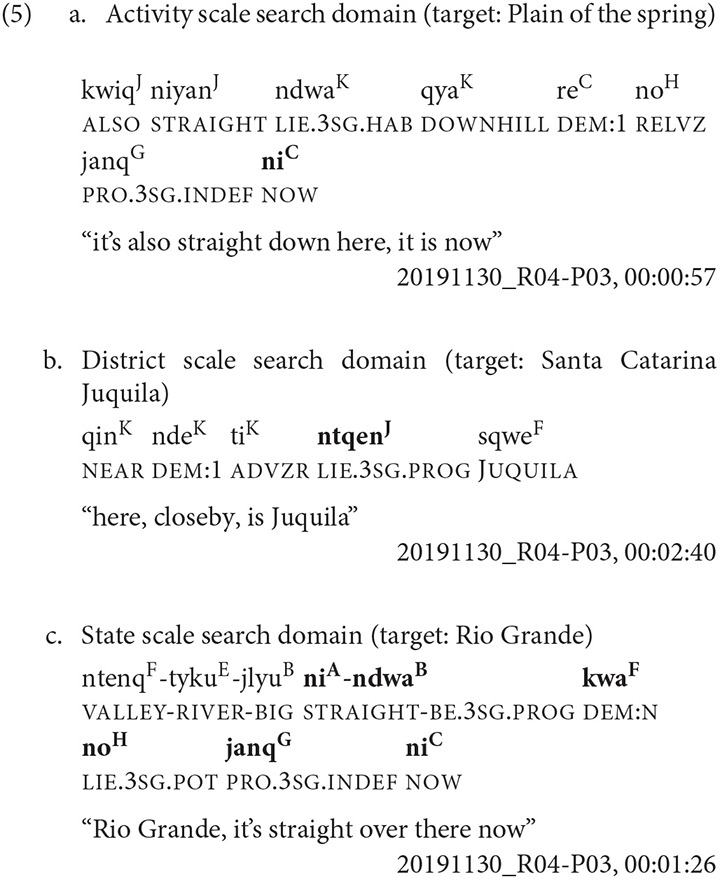


Importantly, the pattern we see with manual pointing is quite distinct from the pattern with demonstrative choice. At larger scales, the two demonstrative forms are used with near-equal frequency, suggesting that factors other than distance exercise a greater influence at those scales. By contrast, manual points are produced with a raised elbow significantly more often at larger scales. Thus, pointing form provides cues to the scale of the search domain in a way that demonstrative form does not.

#### 4.3.3. Distance and Scale Influence How Demonstratives and Points Are Co-organized

One phenomenon that we investigated showed distance effects at all scales. This was the co-organization of demonstrative forms and pointing types.

When speakers used a demonstrative expression with a chin point, they showed a marginal preference for the neutral demonstrative form. This preference was influenced by target distance: the farther the target from the speech dyad, the stronger the preference. It was also influenced by target scale, as the trend was weaker at the largest of the study scales. In a notable contrast, when speakers paired a demonstrative with a manual point, they showed a strong preference for using a speaker-proximal demonstrative form. Again, this preference was influenced by target distance: the farther the target from the speech dyad, the stronger the preference. In this case, the preference grew stronger as the scale size increased. We illustrate these findings in the following examples.

**Figure 7:**
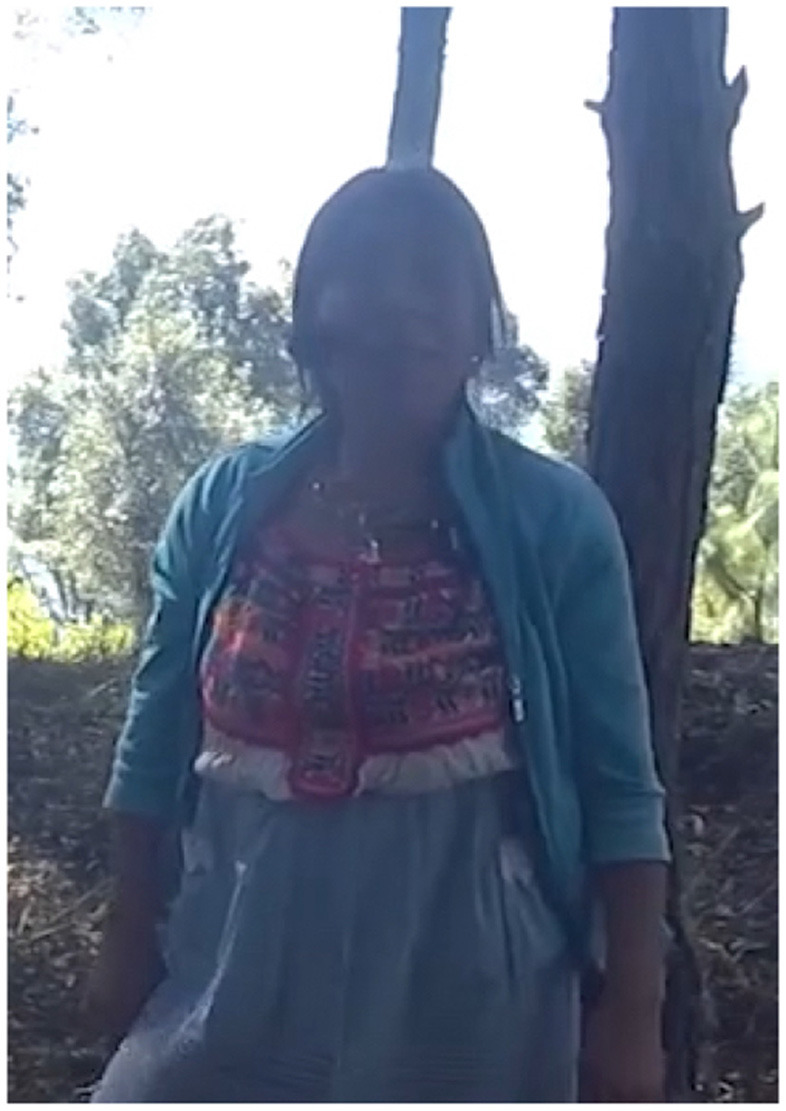
Dem. + chin point [target: Oaxaca].

**Figure 8:**
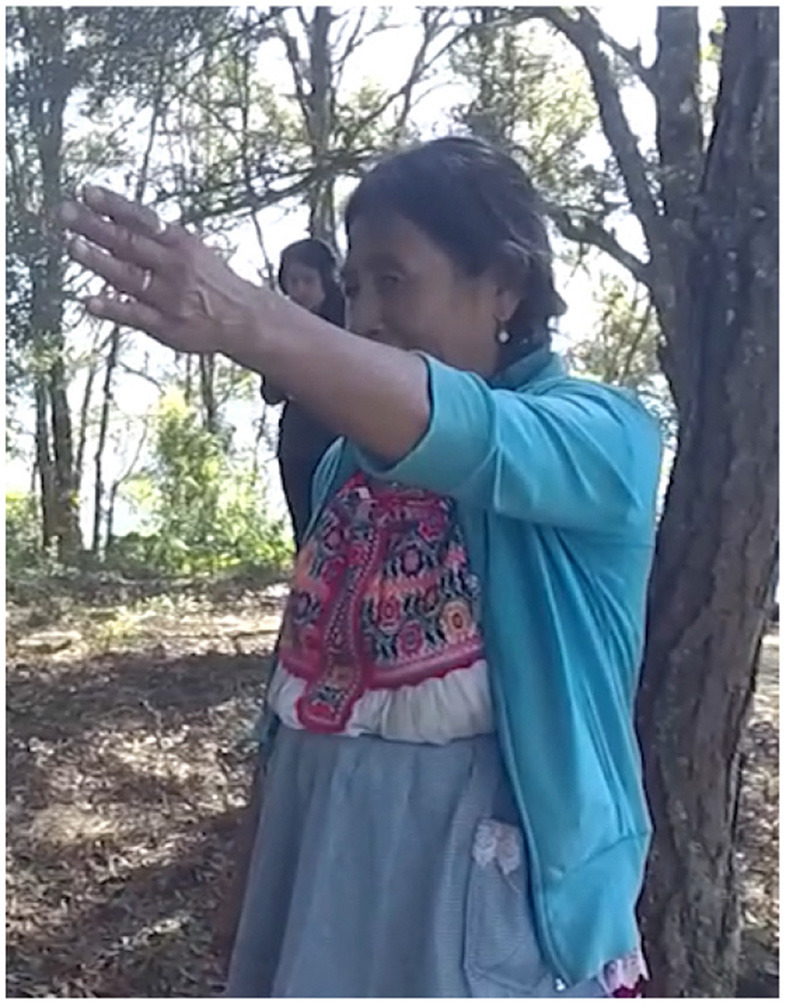
Dem. + manual point [target: Oaxaca].

In Example 6, the speaker is standing at the Petition Monument, the fifth stop on the kyqya^C^ kcheq^B^ trail. She indicates the city of Oaxaca, a state scale target, using a demonstrative with a chin point, and uses the neutral demonstrative form, *kwa*^*F*^ (Example 6a, Figure 7). When indicating the same state scale target using both a demonstrative and a manual point, she uses the speaker-proximal demonstrative form, *nde*^*C*^ (Example 6b, Figure 8).


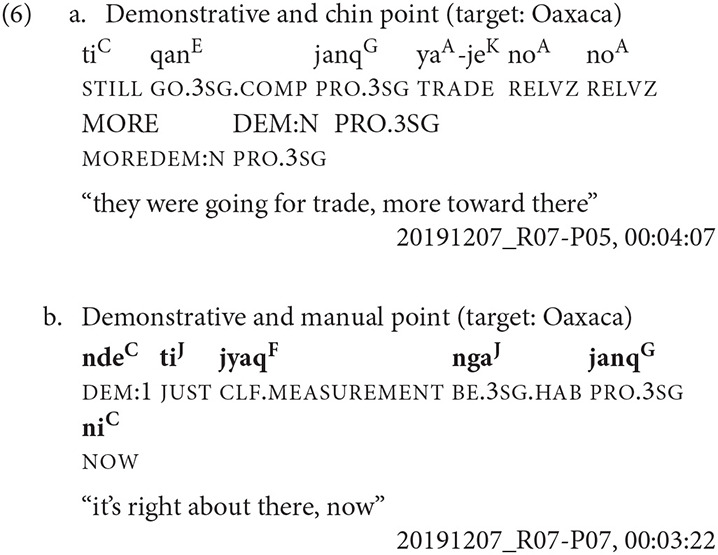


At least one of the above patterns of co-organization has a parallel in smaller-scale space. In studies conducted in laboratory environments, speakers of Dutch and of American English showed a preference for pairing (speaker or speech dyad)-proximal demonstrative forms with manual points (Piwek et al., 2008; Cooperrider, 2011, 2015), though in the study where target distance was explored as a potential conditioning factor (Cooperrider, 2011, 2015) participants showed none of the sensitivity to distance that we see in our study results. In explaining the affinity of proximal demonstratives and pointing, Piwek et al. (2008) and Cooperrider (2011, 2015) focus on the contribution of the demonstrative to the multimodal indicating act, positing that the marked proximal form more “intensely” recruits the attention of the addressee in these constructions. Neither account is explicit about the role of the pointing gesture in these cases of more “intense” multimodal indicating.

Our study results provide a clue to the roles of both the demonstrative and the pointing gesture when they are coupled for more “intense” indicating. At the two largest scales operationalized for the study, we found that demonstratives ceased to participate in a distance-influenced oppositional paradigm, while pointing gestures remained informative about two dimensions of the search domain: its direction and distance. In exactly those contexts, we found the closest relationship between the speaker-proximal demonstratives and the manual point. We propose, in line with Piwek et al. (2008) and Cooperrider (2011, 2015), that in this context the proximal demonstrative is indeed recruiting attention with greater intensity. We further suggest that the demonstrative is orienting visual attention not primarily to the target, but instead (and in some cases exclusively) to the more informative contribution of the speaker's gesturing body (for a similar suggestion, cf. Bangerter, 2004). Demonstratives have been shown to call visual attention to speaker's gestures that represent spatial features of a referent (such as its orientation in space, cf. Emmorey and Casey, 2001; Hegarty et al., 2005). Our findings suggest that demonstratives play a similar role in orienting speaker attention to the gesturing body, as well as the indicated target, during multimodal indicating.

If the speaker-proximal demonstratives draw attention to manual points, what role does the neutral demonstrative play alongside chin points? The picture is less clear here, simply because of the small number of data points we were able to collect and analyze for this study. Chin points have been proposed to occur with neutral demonstrative forms in contexts where the gesture is less informative (Enfield, 2001; Mihas, 2017; Cooperrider et al., 2018). In such contexts, the pointing gesture needs to provide few cues for delimiting the search domain, and the speaker may not expect the addressee to shift their full gaze to the gesturing body and it its attention-directing cues. This may well prompt the speaker to recruit the gaze of the addressee to the gesturing body less intensively. More research about the coordination of demonstratives with chin points will be necessary to further investigate this claim.

## 5. Conclusion

This study has systematically considered the influence of scale on multimodal indicating behaviors, a domain hitherto not investigated. By defining multiple scales within what has previously simply been described as “large-scale” or “geographic-scale” space, we have been able to distinguish between those patterns of indicating that are operational at all scales, and those that are constrained to usage in smaller-scale spaces.

Our first finding—that distance does not straightforwardly account for demonstrative choice, pointing use, or pointing form at larger scales—occasions the question of whether other factors may influence multimodal indicating across scales. More research is called for, in particular into such social-pragmatic factors as the attention of the speech act participants, and their conception of the target as being in or outside of a shared domain of activity.

Our second finding—that some features of the organization of demonstratives and points are present across all scales, and even stronger at larger scales—raises additional questions about how demonstratives and pointing gestures jointly function to manage attention. We have suggested here that manual pointing gestures are the most informative of the indicating behaviors when targets are in large-scale space and have proposed that demonstratives may recruit visual attention to manual and chin points primarily, allowing the points themselves to indicate the target location. This proposal prompts empirical questions about the gaze of the addressee in response to multimodal indicating. It also raises more fundamental questions about the sequencing of demonstratives and pointing gestures at indicating events across scales, as well as about the exact temporal alignment between the modalities, since any theory of their joint function relies on evidence from the temporal coordination of speech and gesture.

The combined findings have broader implications for research on the multimodality of language, as they underscore not only the distinct contributions of speech and gesture to the linguistic composite but also the dynamic nature of their interplay. In exploring how the scale of the search space influences the indicating event, we found yet another source of evidence for the intricate organization of multimodal expressions, and for the tailoring of that organization to specific contexts of language use.

## Data Availability Statement

Raw data underlying the conclusions made in this paper are publicly available in the Lund University Humanities Lab's Corpus Server (http://hdl.handle.net/10050/00-0000-0000-0004-1F68-A@view). Analytical materials, including interview protocols, manuals for data collection and coding, the resulting datasets, and the scripts used to perform the statistical analyses have been made available via the Texas Data Repository (https://doi.org/10.18738/T8/QHMQIY).

## Ethics Statement

The studies involving human participants were reviewed and approved by the Swedish National Ethics Authority, Dnr 2019-04621. Written informed consent for participation was not required for this study in accordance with the national legislation and the institutional requirements. Because much of the population under study for this research is not literate, written consent for study participation and data use was not obtained. Instead, we created video recordings of informed consent being given. Participants whose images appear in this paper gave informed consent for identifiable images of themselves to be published. The consent procedures were approved by the authorities of the San Juan Quiahije municipality, and their approval was recognized by the Swedish National Ethics Authority.

## Author Contributions

KM, MG, and NB contributed to the conception and experimental design of the study. KM and EC performed the experiments, collected the data, and helped to collate the data together with experimental assistants. JW and KM conducted the analyses. All authors contributed to the interpretation of the results and to the writing of the manuscript, and approved the final version of the manuscript for submission.

## Conflict of Interest

The authors declare that the research was conducted in the absence of any commercial or financial relationships that could be construed as a potential conflict of interest.

## Footnotes

^1^In this and all examples to follow, the participant-anchored proximal forms are translated to English as *this* or *here* while the neutral demonstrative is translated as *that* or *there*. We provide these translations to facilitate understanding the sentence meaning, while nevertheless cautioning the reader that there is no translation equivalent for the Quaihije Chatino demonstratives in English.

^2^This strategy was also found in users of San Juan Quiahije Chatino sign language, a young sign language emerging in the Quiahije municipality (Mesh, 2017; Cooperrider and Mesh, in press).

^3^Although palm-up gestures can and often do serve deictic functions, we chose to err on the side of caution when creating our form-based exclusion criteria, and removed all gestures with an upturned palm. Future research will examine the full range of deictic handshapes used by Chatino speakers.

^4^For further evidence that pointing form in San Juan Quihaije is influenced by target distance, and not by target altitude, see (Mesh, submitted).

^5^In this and all other examples in which speech and a pointing gesture overlap, the speech co-occurring with the stroke of gesture appears in boldface type.

## Appendix

### Resúmenes—Gyaq^C^

Cuando los humanos interactúan en el mundo, a menudo orientan la atención de los demás hacia ciertos objetos mediante el uso de expresiones demostrativas y el uso de movimientos de la cabeza, la barbilla, o la mano para señalar dichos objetos. Aunque las estrategias de indicación se han estudiado con frecuencia en entornos de laboratorio, sabemos sorprendentemente poco sobre cómo se utilizan los demostrativos y el señalamiento para coordinar la atención en espacios a gran escala y en contextos naturales. Este estudio investiga cómo los hablantes del chatino de San Juan Quiahije, una lengua indígena de México, señalan y usan demostrativos para dar direcciones a lugares conocidos en múltiples escalas (actividad local, distrito, estado). Los resultados muestran que el uso y la coordinación de demostrativos y el señalamiento cambian a medida que crece la escala del espacio de búsqueda del objetivo. En escalas más grandes, es más probable que los demostrativos y el señalamiento se produzcan juntos, y las dos estrategias parecen manejar diferentes aspectos de la búsqueda del objetivo: los demostrativos orientan la atención principalmente al cuerpo que gesticula, mientras que señalar proporciona claves para reducir el espacio de búsqueda. Estos resultados sacan a luz las distintas contribuciones del habla y el gesto al compuesto lingüístico, al tiempo que ilustran el carácter dinámico de su interacción.

Qan^K^ no^K^ ndywenq^J^ qo^E^ xka^I^ ta^A^ la^E^ nten^B^, tqa^K^ ti^K^ ntsanq^B^ qin^K^ chonq^G^ na^F^ no^A^ ndiya^I^ qo^E^ loga^B^ no^K^ nlo^I^ la^J^ ti^J^ qna^G^. Ndiya^I^ tkwa^J^ niyan^J^ muru^K^ jnya^K^ qin^A^ nten^B^ chaq^F^ qne^J^ kasu^K^ qo^E^ jnyi^A^ qya^H^ ndiya^A^ na^F^ qo^E^ loga^B^: ka^C^ chaq^F^ jlanq^J^ qo^E^ ntykwenq^J^ chaq^F^ (qo^E^ chaq^F^ niyan^K^ no^K^ nde^C^: re^C^ kwa^J^ qo^E^ kwa^F^) qo^E^ ka^C^ chaq^F^ qu^B^ xnyi^K^ in^H^ qo^E^ yanq^C^ an^I^ qo^E^ ta^A^ qo^E^ skwaq^G^ tqwan^J^ an^I^. Lo^A^ ktyi^C^ no^E^ nde^C^ in^H^, wa^G^ re^C^ ntsaq^B^ wa^G^ qwan^K^ niyan^K^ nlyaq^I^ no^A^ nga^J^ nten^B^ no^K^ ndywiq^A^ chaq^F^ jnya^J^ chaq^F^ qo^E^ qwan^K^ niyan^K^ ntqu^K^ qo^E^ ntsaq^B^ renq^K^ qan^K^ no^K^ jnya^H^ renq^J^ qin^A^ chaq^F^ jnyi^J^ qya^H^ qo^E^ ktsaq^B^ ndiya^A^ loga^B^ no^K^ nlo^I^ qin^A^ kchin^A^ tyi^A^. Ntqu^B^ wa^G^ chaq^F^ nten^B^ nlo^J^ swi^H^ chaq^F^ qo^E^ qwi^A^ ska^A^ niyan^J^ qne^E^ qan^K^ no^K^ ntsaq^B^ qo^E^ ntqu^B^ ska^K^ na^F^ qo^E^ loga^B^. Nlo^J^ swi^H^ chaq^F^ qo^E^ qwi^A^ ska^A^ niyan^J^ qne^E^ kanq^G^ chaq^F^ ntqu^B^ ran^H^ si^K^ ta^A^ tyjyuq^A^ nga^J^ qo^E^ ntqen^A^ loga^B^ no^K^ nda^E^ renq^J^ qo^E^ ndywiq^I^ renq^A^ chaq^F^ qin^J^ sqen^A^ no^A^ ntqen^A^ renq^A^.

### 1. Chatino Orthography

#### 1.1. Practical Orthography: Correspondences With the International Phonetic Alphabet

We use a practical orthography to transcribe Quiahije Chatino, rather than International Phonetic Alphabet (IPA). The consonant phonemes, in practical orthography and IPA, are as follows: bilabial p = [p], b = [nb], m = [m]; apicodental t = [t], d = [nd], ts = [ʦ],s = [s], n = [n], r =[ɾ], l = [l]; laminoalveolar ty = [t̻ ], ny = [n̻], ly = [l̻ ]; alveolopalatal ch = [ʧ], x = [ʃ], y = [j]; Velar k = [k], g = [ng]; labiovelar w = [w]; glottal q = [ʔ], j = [h]. The consonant phonemes, in practical orthography and IPA, are as follows: /i/, /u/, /e/, /o/, /a/. Where the IPA represents nasalized vowels with a diacritic, as in /ã/, we use a vowel followed by an n, as in /an/.

#### 1.2. Transcription of Tone Values

Every word in Quiahije Chatino bears one tone. The tone is represented as a capital letter at the end of the word. The tone value assignments are: A = [Low], B = [High-Low], C = [Mid], E = [High], F = [Low-Mid], G = [Low-High], H = [Mid-superhigh], I = [Midhigh], J = [Mid-Low], K= [superhigh], L = [Low superhigh], and M = [superhigh Low].

### 2. Distribution of Indicating Strategies and Demonstrative Forms, Across Targets, and Across Participants

#### 2.1. Demonstrative Forms (nde^C^/re^C^ = Proximal, kwa^F^ = Neutral) by Target

**Table:**

**Scale**	**Target, Quiahije Chatino**	**Target, Translated**	**nde^C^/re^C^**	**kwa^F^**
Activity	ntenq^F^ tiyuq^G^	Plain of the Spring	21	24
	tu^C^ kchi^C^	Mountain Pass	21	10
	ke^A^ tiyeq^B^	Sour Rock	22	11
	ke^A^ ku^E^ suq^C^	Turkey Breast Rock	22	17
	lo^A^ si^K^ kyqya^C^ kcheq^B^	Petition Monument	30	6
	kyqya^C^ kcheq^B^	Peak of Thorn Mountain	43	8
District	kchin^A^	San Juan Quiahije	17	6
	ntenq^F^	Cieneguilla	20	19
	tqwa^A^ tyku^E^	San José Ixtapan	19	20
	skwi^E^	San Miguel Panixtlahuaca	34	18
	sqwe^F^	Santa Catarina Juquila	38	10
	ke^G^ xin^E^	Santiago Yaitepec	16	21
State	se^A^ na^A^ nya^K^	Santiago Minas	18	17
	tsi^C^	San Marcos Zacatepec	16	12
	ntenq^F^ tyku^E^ jlyu^B^	Rio Grande	9	15
	lo^A^ ntqa^B^	Oaxaca City	19	22

#### 2.2. Indicating Strategies (dem. Alone, dem. + Manual Point, dem. + chin point) by Target

**Table:**

**Scale**	**Target, Quiahije Chatino**	**Target, Translated**	**D**	**DM**	**DC**
Activity	ntenq^F^ tiyuq^G^	Plain of the Spring	22	18	5
	tu^C^ kchi^C^	Mountain Pass	21	9	1
	ke^A^ tiyeq^B^	Sour Rock	22	8	3
	ke^A^ ku^E^ suq^C^	Turkey Breast Rock	27	9	3
	lo^A^ si^K^ kyqya^C^ kcheq^B^	Petition Monument	25	10	1
	kyqya^C^ kcheq^B^	Peak of Thorn Mountain	36	15	0
District	kchin^A^	San Juan Quiahije	9	11	3
	ntenq^F^	Cieneguilla	36	15	0
	tqwa^A^ tyku^E^	San José Ixtapan	18	21	0
	skwi^E^	San Miguel Panixtlahuaca	29	19	4
	sqwe^F^	Santa Catarina Juquila	21	26	1
	ke^G^ xin^E^	Santiago Yaitepec	12	21	4
State	se^A^ na^A^ nya^K^	Santiago Minas	15	20	0
	tsi^C^	San Marcos Zacatepec	15	13	0
	ntenq^F^ tyku^E^ jlyu^B^	Rio Grande	9	11	4
	lo^A^ ntqa^B^	Oaxaca City	16	22	3

#### 2.3. Demonstrative Forms (nde^C^/re^C^ = Proximal, kwa^F^ = Neutral) by Participant

**Table:**

**Participant**	**nde^C^/re^C^**	**kwa^F^**
R04	65	25
R05	47	46
R06	24	27
R07	35	38
R08	59	43
R09	64	26
R10	43	14
R11	28	17

#### 2.4. Indicating Strategies (dem. Alone, dem. + Manual Point, dem. + Chin Point) by Participant

**Table:**

**Participant**	**D**	**DM**	**DC**
R04	35	52	3
R05	38	52	3
R06	23	18	10
R07	36	35	2
R08	51	49	2
R09	58	30	2
R10	47	6	4
R11	22	14	9

### 3. Regression Models

#### 3.1. Analysis 1: Outcome: Choice of Demonstrative, Predictors: Scale and Distance

**Table:**

	**Estimate**	**Std. Error**	***z* value**	**Pr(>|z|)**
(Intercept)	1.598	0.262	6.109	0.000
distance.rescaled	−2.833	0.520	−5.452	0.000
scale.factordistrict	−0.809	0.371	−2.182	0.029
scale.factorstate	−1.576	0.322	−4.901	0.000
distance.rescaled:scale.factordistrict	2.222	0.706	3.150	0.002
distance.rescaled:scale.factorstate	2.577	0.659	3.909	0.000

#### 3.2. Analysis 2: Outcome: Chin Point, Predictors: Scale and Distance

**Table:**

	**Estimate**	**Std. Error**	***z* value**	**Pr(>|z|)**
(Intercept)	−3.115	0.517	−6.032	0.000
distance.rescaled	0.437	0.885	0.494	0.622
scale.factordistrict	0.982	0.659	1.491	0.136
scale.factorstate	−0.252	0.713	−0.353	0.724
distance.rescaled:scale.factordistrict	−1.831	1.316	−1.392	0.164
distance.rescaled:scale.factorstate	0.505	1.236	0.408	0.683

#### 3.3. Analysis 3: Outcome: Manual Point, Predictors: Scale and Distance

**Table:**

	**Estimate**	**Std. Error**	***z* value**	**Pr(>|z|)**
(Intercept)	−1.555	0.328	−4.747	0.000
distance.rescaled	1.606	0.487	3.296	0.001
scale.factordistrict	1.568	0.363	4.317	0.000
scale.factorstate	1.452	0.321	4.526	0.000
distance.rescaled:scale.factordistrict	−1.810	0.683	−2.653	0.007
distance.rescaled:scale.factorstate	−1.449	0.642	−2.258	0.024

#### 3.4. Analysis 4: Outcome: Elbow Height, Predictors: Scale and Distance

**Table:**

	**Estimate**	**Std. Error**	***z* value**	**Pr(>|z|)**
(Intercept)	−1.481	0.589	−2.513	0.012
distance.rescaled	1.985	0.907	2.187	0.029
scale.factordistrict	1.476	0.616	2.398	0.017
scale.factorstate	2.712	0.626	4.333	0.000
distance.rescaled:scale.factordistrict	−1.772	1.151	−1.539	0.124
distance.rescaled:scale.factorstate	−2.404	1.132	−2.125	0.034

#### 3.5. Analysis 5: Outcome: Head Point, Predictors: Scale and Demonstrative

**Table:**

	**Estimate**	**Std. Error**	***z* value**	**Pr(>|z|)**
(Intercept)	−3.371	0.529	−6.376	0.000
dem.factorkwaF	0.912	0.582	1.566	0.117
scale.factordistrict	0.093	0.586	0.159	0.873
scale.factorstate	0.660	0.669	0.987	0.324
dem.factorkwaF:scale.factordistrict	0.014	0.803	0.018	0.985
dem.factorkwaF:scale.factorstate	−1.412	0.987	−1.431	0.152

#### 3.6. Analysis 6: Outcome: Manual Point, Predictors: Scale and Demonstrative

**Table:**

	**Estimate**	**Std. Error**	***z* value**	**Pr(>|z|)**
(Intercept)	−0.980	0.331	−2.957	0.003
dem.factorkwaF	−0.430	0.330	−1.305	0.192
scale.factordistrict	1.240	0.257	4.836	0.000
scale.factorstate	1.666	0.346	4.815	0.000
dem.factorkwaF:scale.factordistrict	−0.488	0.433	−1.128	0.259
dem.factorkwaF:scale.factorstate	−0.955	0.515	−1.867	0.063
