# Supplementary material for: Effects of Scale on Multimodal Deixis: Evidence From Quiahije Chatino
Source: Front Psychol. 2021 Jan 12;11:584231. doi: 10.3389/fpsyg.2020.584231 (PMC7835423; doi:10.3389/fpsyg.2020.584231)
Supplement: Supplementary file 1 [file Data_Sheet_1.PDF]

Target	Dem kwaF	ndeC/reC
kchinA	6	17
keA kuE suqC	17	22
keA tyeqB	11	22
keG xinE	21	16
kiqiyaC kcheqB	8	43
loA ntqaB	22	19
loA siK kiqiyaC kcheqB	6	30
ntenF	19	20
ntenF tykuE jlyuB	15	9
ntenF tyuqG	24	21
seA naA nyaK	17	18
skwiE	18	34
sqweF	10	38
tqwaA tykuE	20	19
tsiC	12	16
tuC kchiC	10	21

Supplementary Table 1. Demonstrative forms (kwaF = neutral, ndeC/reC = speaker-proximal) by target

Target	Ind_Strategies D	DC	DM
kchinA	0	3	11
keA kuE suqC	0	3	9
keA tyeqB	0	3	8
keG xinE	0	4	21
kiqiyaC kcheqB	0	0	15
loA ntqaB	0	3	22
loA siK kiqiyaC kcheqB	0	1	10
ntenF	0	3	23
ntenF tykuE jlyuB	0	4	11
ntenF tyuqG	0	5	18
seA naA nyaK	0	0	20
skwiE	0	4	19
sqweF	0	1	26
tqwaA tykuE	0	0	21
tsiC	0	0	13
tuC kchiC	0	1	9

Supplementary Table 2. Indicating strategies (plain demonstrative, dem + chin point, dem + manual point) by target

Participant	Dem	
	kwaF	ndeC/reC
R04	25	65
R05	46	47
R06	27	24
R07	38	35
R08	43	59
R09	26	64
R10	14	43
R11	17	28

Supplementary Table 3. Demonstrative forms (kwaF = neutral, ndeC/reC = speaker-proximal) by participant

Participant	Indicating_Strategies		
	D	DC	DM
R04	0	3	52
R05	0	3	52
R06	0	10	18
R07	0	2	35
R08	0	2	49
R09	0	2	30
R10	0	4	6
R11	0	9	14

Supplementary Table 4. Indicating strategies (plain demonstrative, dem + chin point, dem + manual point) by participant
