# Supplementary figures and images for: Effects of Scale on Multimodal Deixis: Evidence From Quiahije Chatino
Source: Front Psychol. 2021 Jan 12;11:584231. doi: 10.3389/fpsyg.2020.584231 (PMC7835423; doi:10.3389/fpsyg.2020.584231)

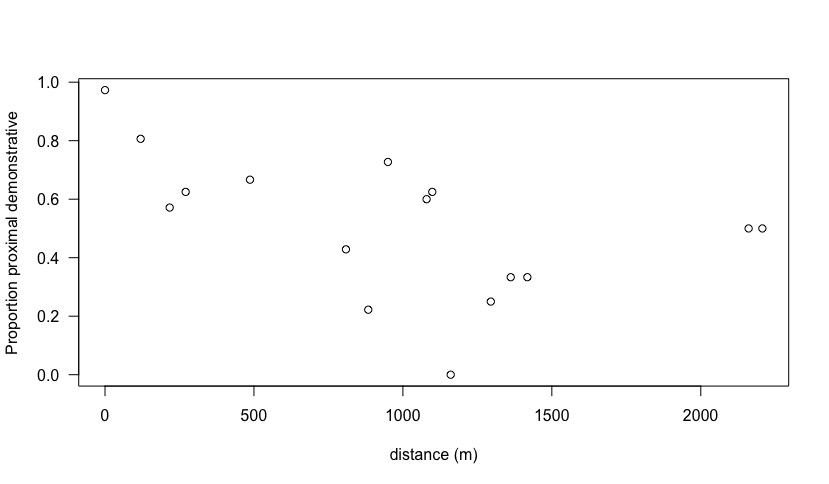

Supplement: Supplementary file 2 [file Image_1.JPEG]
